# Experiencing COVID-19 Through the Patient Lens to Promote Empathy: Pilot Testing a Virtual Reality Learning Opportunity

**DOI:** 10.1177/23743735241241462

**Published:** 2024-04-25

**Authors:** Heather Thomson, Lisa Di Prospero, Sarah Xiao, Laurie Legere, Tamara Harth, Laura Rashleigh, Maria Parzanese, Lorraine Graves, Kyle Wilcocks, Fahad Alam

**Affiliations:** 1 70379 Lawrence Bloomberg Faculty of Nursing, University of Toronto, Toronto, ON, Canada; 2 Practice-Based Research and Innovation, 71545 Sunnybrook Health Sciences Centre, Toronto, ON, Canada; 3 Department of Radiation Oncology, 12366 Temerty Faculty of Medicine, University of Toronto, Toronto, ON, Canada; 4 Department of Anesthesiology & Pain Medicine, Temerty Faculty of Medicine, Toronto, ON, Canada; 5 Richmond, British Columbia, Canada

**Keywords:** virtual reality, education, COVD-19, empathy

## Abstract

Understanding the patient's experience with COVID-19 was essential to providing high-quality, person-centered care during the pandemic. Having empathy or being able to understand and respond to the patient's experience may lead to improved outcomes for both patients and clinicians. There is mixed evidence about how best to teach empathy, particularly related to promoting empathy during COVID-19. Literature suggests that virtual reality may be effective in empathy-related education. In collaboration with four patient partners with lived experience, a 360° VR video was developed reflecting their stories and interactions with the healthcare system. The aim of this study was to pilot test the video with interprofessional healthcare providers (HPs) to explore acceptability and utility, while also seeking input on opportunities for improvement. Eleven HPs reviewed the video and participated in one of three focus groups. Focus group data were analyzed using thematic analysis. Data suggest that video content is acceptable and useful in promoting a better understanding of the patient's experience. Building on these encouraging findings, additional iterations of videos to promote empathy will be developed and tested.

## Key Points

1. Empathy among healthcare providers (HPs) contributes to the provision of high-quality patient care.
2. Virtual reality (VR) is one approach that has been used to promote the development of empathy among HPs.
3. A 360° VR video co-designed with patient partners is an acceptable and useful tool in promoting an understanding of the patient's experience with COVID-19, particularly during the early phases of the pandemic.
4. Further research regarding outcomes associated with the use of VR to promote empathy is required.

## Background

Empathy is a multidimensional concept which involves understanding the experiences of others, feeling, and sharing others’ emotions, and acting upon and responding to those feelings.^1,2^ Bertrand ^3^ and colleagues suggest empathy allows individuals to “learn from others’ pain and to know when to offer support” (p.1), providing opportunities for a compassionate response.^4^ Empathy is essential for positive interpersonal interactions, creating connections and contributing to our sense of society.^4^ In healthcare, empathy is fundamental to patient care,^5^ associated with positive outcomes for patients and healthcare providers (HPs).^6–9^

At the height of COVID-19, clinical practices had to shift and adapt to the new realities of the global pandemic, including the use of virtual care, limiting visitors, full personal protective equipment requirements, among others.^10^ While these changes had an impact on the health system, organizations, and HPs, they ultimately impacted the patient's experience. For example, the inability to see providers’ faces may have hindered human connection and development of the therapeutic relationship.^10^ During the early stages of the pandemic, patients reported increased loneliness related to isolation and visitor restrictions,^10,11^ as well as distress, fear, uncertainty, and worry.^2,11^ Given these negative experiences, the provision of “humanistic care based on a mutual understanding” (p.8)^12^ between patients and HPs was essential in providing optimal patient care.^12^

Literature suggests that empathy levels decline during health professions education.^4,13^ Post-graduation, there is also variation in empathy across disciplines and HPs.^2,7^ Barriers to empathy include lack of empathy-related education as part of both undergraduate and continuing education, in addition to increased workload and decreased time.^6^ While empathy is integral to high-quality care, clinicians may find it difficult to empathize with conditions they have not experienced first-hand.^14^ Given that little was known about COVID-19 during the first wave of the pandemic and many HPs had not personally experienced COVID-19 infection, it was important to consider how best to promote an understanding of this experience.

Strategies used in empathy-related education include face-to-face training, role-playing, videos, and other types of experiential and immersive learning such as virtual reality (VR).^8,15^ VR has been recognized as a method to promote empathy by allowing users to feel as though they are in the world of another.^5,16,17^ VR provides an “effective medium for interactive storytelling,” (p. 64)^17^ allowing the user to embody another's experience and understand it from their perspective.^3,17^ VR enables perspective taking, giving viewers the ability to imagine what it is like to be the other, increasing empathic response.^3^ This paper reports on the development and pilot testing of a VR video aimed at promoting an understanding of patients’ lived experiences with COVID-19 among HPs.

## Methods

### Recruitment of Patient Partners & Video Development

This project was guided by a co-design approach where patients with lived experience were invited to participate as partners in the development and production of the VR video. Four patient partners responded to the invitation to participate, circulated using hospital ads and social media. A $25 gift card was provided as a token of appreciation for participation. Best practices for patient partnership suggest that there should be more than one patient; however, there are no consistent guidelines for the optimal number of patients to include on research teams.^18^ While our initial aim was to recruit up to 10 patients, we had a low response rate, possibly because recruitment took place in the summer of 2020 as the first wave of the pandemic stabilized.

To develop the video script, patient partners were invited to share their stories in an online focus group. Based on these stories, the research team developed a draft script, including five vignettes representing the patient's journey from diagnosis to follow-up care. In keeping with best practices for confirming qualitative data,^19^ themes and the script were shared with patient partners, and they participated in further development and editing to accurately reflect their experiences. The VR video was filmed using 360° video and produced and edited with input from the patient partners.

### Study Design

The VR video was pilot tested using a qualitative descriptive study design. HPs were invited to view the video and share their feedback in semi-structured focus groups, guided by open-ended questions to explore utility and acceptability (Table 1).

**Table 1.** Focus Group Questions.

What is your initial impression of the video? What are your thoughts on the content of the video? How do you feel about the mode of delivery? Would you say that the video could be used to provide you with an understanding of what it is like to be a patient experiencing COVID-19? Do you think that watching the video will have an impact on the care that you provide to COVID-19 patients? Why or why not? If this video were implemented in practice, how would you see it being used? Do you have any specific feedback or suggestions on how to improve the video? Is there anything else that you would like to share about your experience with the video?

### Focus Group Participants

Focus group participants were recruited from the hospital using email advertisements. Eleven interprofessional HPs agreed to participate and provided written informed consent. A $25 gift card was provided as a token of appreciation.

### Methods and Analysis

Participants joined one of three virtual focus groups. Prior to focus group sessions, participants were asked to watch the video using a computer, tablet, or mobile device. They were also offered cardboard VR goggles that could be used with a mobile phone for a more immersive experience. To assess acceptability, participants were invited to share their perceptions of video content and mode of delivery.^20^ Utility was assessed by inviting participants to reflect on whether the video resulted in a better understanding of the patient's experience. Participants were also asked to share opportunities to improve future iterations.

With consent, focus groups were digitally recorded and transcribed verbatim. Data were analyzed using thematic analysis.^22^ Focus group transcripts were independently reviewed and analyzed by four researchers, beginning with in-depth reading, followed by coding, and grouping codes into potential themes. After achieving consensus, themes were finalized, and representative exemplars were selected.

## Results

Five themes were identified: realistic representation of COVID-19 experience, teaching tool for different audiences, impact on clinicians, perspectives on VR, and opportunities for improvement and further development (Table 2).

**Table 2.** Quotes to Illustrate Identified Themes.

Theme	Illustrative Quotes
Realistic Representation of the COVID-19 Experience	“I would say that I thought it was pretty realistic in terms of patient experience, like from the other end… I thought it resonated well in terms of communicating what it would be like to be in an emergency department with covid but not have a positive test result and therefore, you know how you’re dealt with…” (FG 1). “I also think it's kind of reminiscent of what patients describe to us in terms of, you know, there's so much coming at me, like I can't focus on anything. So I thought that was pretty realistic.” (FG 2) “I have a couple of close friends who actually worked in the ICU at the beginning and throughout, and I would say that from what they've told me, this is kind of like the same side of things.” (FG 2)
Teaching Tool for Different Audiences	“…the learnings from this video are really applicable to any situation across health care.” (FG 1) “I think it would be for health care professionals very beneficial to watch it, not on the not on their own, so not as ‘ok, this is a learning module you have to do so’, you just do it by yourself and you go on just to get it done. But rather as ‘OK, we're going to devote an hour of our unit staff meeting this month to looking at this and discussing it afterwards’…” (FG 1) “So, I mean, I think it forces people to reflect on how you should maybe be engaging with people when you're communicating…” (FG 2) “I think it was well done if the vehicle for it is to be teaching video to health care providers to see the overall difference and to see things from the patient's side” (FG 3)
Impact on Clinicians	“Again, never having gone through it or encountered someone directly. There were times when I felt like I was, I came close to feeling like I was almost living it and it was frightening” (FG 1). “So this was really eye-opening for me, you know, I've read papers and I've listened to the news and a few friends of mine have even had it. But actually seeing this, the simulation of the breathing difficulties, etc., and it being from the patient's perspective, I thought was impactful, like visually from that perspective.” (FG 1) “I get into stuff like this, very scary and now other people may not take that in the way I do, but I think the more you can promote that emotional response, I think the more impactful it will be.” (FG 1). “Yeah, I it had a great impact on me, actually, the feelings that patients had. I have always … had this fear of covid, but never, never experienced anything like that. But the video and I watched it with the … virtual reality camera [goggles] and [it] had a really great impact, and how I felt about covid, really it was worse than what I thought it would be.” (FG 2) “So if somebody is trying to learn how to deal with patients with covid, then understanding what it was like to be completely isolated, to not know if you were going to die and not see your family and then worrying about everyone else and then having nobody to help at home. I mean, it was a terrible situation for a lot of people.” (FG 3).
Perspectives on Virtual Reality	“… I just watched it again now I did think it [VR] helped a bit” (FG 1) “I watched it with the virtual reality camera [goggles] and it had a really great impact” (FG 2) “I like the style of the actual shooting of the video, but it was from her eyes looking out both at home and in the ER bed.” (FG 3) “Is the virtual piece really anything that's necessary in delivery of this teaching moment and this message? Like to be able to look around the room, what does that add to your perception of the situation and how she was treated or mistreated? Really? So, I would just put that out there for some thought provoking, like is the virtual piece really, is it a distraction?” (FG 3)
Opportunities for Improvement & Further Development	“I still thought it could have gone further. So she made the connection. She listened. She validated. But to me, I'm a kind of a next steps person. So, if I've now got chronic covid, what do I do? OK, well, we don't have all the answers, I know, but I'm the kind of person I need a plan. So help me build the plan. What I might expect in terms of physical activity, is there any other medications I can take?” (FG 1) “I think that it would be a little bit more realistic if the placements had been different, like if you are sick with congestion, like the person who's speaking is going to sound a lot louder to themselves. And given that there's all the gowning, the mask and the face shield, the person who's talking is going to sound a lot lower than they normally do, whereas the video did exactly the opposite. So I think that would be one way to improve it” (FG 2)

Theme 1: Realistic Representation of COVID-19. Participants reported that the video was realistic, reflecting personal experiences, as well as anecdotal experiences shared by friends, family, and colleagues. This was a particularly important finding as many HPs had not yet personally experienced COVID-19.

Theme 2: Teaching Tool for Different Audiences. The video could be used as a teaching tool to promote understanding of the patient's experience across a range of audiences and regarding the healthcare system more generally. Participants identified the importance of having a group discussion or debriefing following the video to further embed learning and behavior change.

Theme 3: Impact on Clinicians. Participants described the video as allowing them to feel like they were in the patient's shoes. Findings suggest that the video evoked a broad range of feelings including fear, uncertainty, sadness, and frustration among viewers. Specific questions to elicit how the video worked and subsequent impacts on clinician behavior were not included in the focus group guide; however, participants provided some information that may help in understanding how the video worked to promote empathy. They highlighted the importance of creating an emotional response to the video, while generating an understanding of the unique situations experienced by patients.

Theme 4: Perspectives on VR. One participant elected to use the VR goggles, with most opting to watch the video on a computer. The participant who used the VR goggles described them as being useful and contributing to the experience, whereas those who watched using a computer appreciated the ability to pan around the room and view the scenarios through the patient's eyes. However, there were questions about whether the expanded capabilities of the VR video enhanced or distracted from the overall effect.

Theme 5: Opportunities for Improvement and Further Development. Although feedback about the video was overwhelmingly positive, suggestions from participants included improving technical components such filming and sound logistics, as well as incorporating additional perspectives and next steps for providers to consider. Moreover, participants noted there might be an expanded role of patient experts in the development of empathy curriculum across all health professions, both pre- and post-licensure.

## Discussion

Focus group data suggest the video was realistic and impactful, conveying a sense of being in the patient's shoes. Using 360° video enhanced with auditory effects such as internal dialogue, breath and heartbeat sounds, participants were able to feel as though they were immersed in the patient's world. This is congruent with literature which suggests that these features may enhance the illusion of embodying another.^3,21^ The video further highlighted the need for open dialogue between patients and HPs, even in situations when there are unknown answers or prognoses. While the video prompted reflection on how to improve care, opportunities to enhance participant learning were identified, such as providing structured group dialogue and debriefing.^22^ In addition to strengthening learning, debriefing helps to consolidate knowledge, promote self-reflection, and may provide support for feelings of distress and helplessness elicited by the video.^5,23^

Overall, focus group findings suggest that the video is both acceptable and useful. As this pilot project was exploratory in nature, further research is required to determine whether the video results in a measurable change in empathy levels and to assess the effectiveness of combining VR with other educational supports such as debriefing. To date, VR has often been evaluated with a focus on usability rather than learning outcomes.^23^ While there are studies documenting the use of VR to promote empathy,^24,25^ none were identified that explore empathy related to COVID-19.

Given these positive preliminary findings, there are additional opportunities to test the use of VR to promote an understanding of different disease conditions, particularly those where assumptions or bias are prevalent, such as fibromyalgia, chronic pain, and substance use disorder. The team is actively exploring opportunities to integrate this type of learning in both pre- and post-licensure education.

### Limitations

A major limitation of this study was the small sample of participants who used the VR goggles. Given that only one participant viewed the video with goggles, it is unknown whether goggles enhanced the experience. In VR, immersion occurs when participants’ awareness of time and the real world become disconnected, allowing them to be present in the VR world.^23^ A sense of immersion generated using technology such as head-mounted devices (HMDs) may be more effective in promoting empathy.^16^ The use of immersive HMD may also lead to increased engagement and skill acquisition.^26^

Two-dimensional (2D) VR using a desktop computer or flat screen is considered a non-immersive form of VR.^23^ While varying levels of immersion may have affected participants’ perceptions of the video, those who used a 2D viewing method did report an increased understanding of the patient's perspective. Further research into the role of immersion in healthcare-related VR is required.

## Conclusion

Findings suggest that the VR video may have value as a teaching tool in promoting empathy among HPs. By providing clinicians with an understanding of the patient's experience with COVID-19, it is anticipated that they will be more attuned to patient needs and preferences, resulting in higher quality, patient-centered care.
